# Prognosis in flail upper limbs

**DOI:** 10.1186/1753-6561-9-S3-A21

**Published:** 2015-05-19

**Authors:** Anil Bhatia

**Affiliations:** 1 Joshi Hospital, Pune, Maharashtra 411004, India

## 

These patients have suffered complete disconnection of the upper limb from the brain and spinal cord. Restoration of function involves reinnervation of muscles for control of the shoulder, the elbow and of the hand. The preponderance of root avulsions implies that there is a serious deficiency of donor nerves. Traditional nerve transfers using the spinal accessory nerve and the phrenic or the intercostal nerves provide consistent results for the supraspinatus and biceps muscles. However, this merely enables the patient to place the arm in space, which can be made more useful as a paperweight by secondary operations such as derotation osteotomy of the humerus and wrist fusion in pronation. Addition of thoraco-brachial grasp demands reinnervation of the pectoralis major using an intercostal. Systematic exploration of the brachial plexus is essential as ruptured root stumps in the neck are invaluable sources of growing axons.

The real stumbling block is restoration of hand function. The sparse axon content of the intercostal nerves is insufficient for the median nerve. Incidence of extra-foraminal lesions varies. Bertelli has reported 1 or 2 roots being available for grafting in 80% of patients. My own experience does not match this figure. Grafting from the roots to the median nerve has produced flexion of the fingers in around 40% of cases. However, this is usually feeble. A part of the force is spent in flexion of the wrist. Wrist fusion helps in strengthening the finger flexion. However, the patient cannot open the fist. Complicated actions in self-care, leisure activities and at work are not possible. Traditional methods of nerve reconstruction can, thus, offer only primitive use of the paralysed upper limb.

Introduction of the contralateral C7 raised hopes of better distal function. However, experience with transfer has been unsatisfactory in terms of the strength recovered, and the success rate. The need for synchronous activity of the donor upper limb to activate the paralysed hand interferes with independent use. The remarkable improvement reported by Wang seems to indicate that shortening the gap between the donor and target nerves is responsible for this change in the achieved result. We shall have to await our own results with this technique before commenting on the utility of this strategy.

In this context, we must discuss the double free functioning muscle transfer strategy. The available nerves (spinal accessory and intercostals) are harnessed for flexion and extension of the elbow and flexion and extension of the fingers. However, the number of surgical procedures involved increases the delay till the final result is achieved. In addition, lack of biofeedback from the transferred muscle implies that prolonged training is necessary for effective use of the recovered functions. Most often, grasp and release is possible only with constant use of a splint. Hence, very few units have adopted Doi’s elaborate plan.

Variations on this have been proposed by Tu (combined adductor longus+gracilis transfer in first stage followed by a second free gracilis transfer) and by Shin and Alan Bishop (one stage re-innervation of biceps using intercostals, a free gracilis for finger flexion using intercostals and of triceps using the spinal accessory nerve). There are no detailed reports of the results achieved.

To summarise, independent handling of objects is still a target to be achieved. Control of the shoulder and elbow is consistently obtained. Finger flexion of a reasonable strength, too, is within reach.

